# Application of X-Ray Computed Tomography to Identify Defects in Lost Wax Ceramic Moulds for Precision Casting of Turbine Blades

**DOI:** 10.3390/ma17205088

**Published:** 2024-10-18

**Authors:** Krzysztof Żaba, Dawid Gracz, Tomasz Trzepieciński, Marzanna Książek, Ryszard Sitek, Adam Tchórz, Maciej Balcerzak, Daniel Wałach

**Affiliations:** 1 Department of Metal Working and Physical Metallurgy of Non-Ferrous Metals, Faculty of Non-Ferrous Metals, AGH University of Krakow, Al. Adama Mickiewicza 30, 30-059 Cracow, Poland; balcerzak@agh.edu.pl; 2 Aptiv Services Poland S.A., ul. Podgórki Tynieckie 2, 30-399 Cracow, Poland; dawid1994g@gmail.com; 3 Department of Manufacturing Processes and Production Engineering, Rzeszów University of Technology, 8 Powstancow Warszawy Ave., 35-959 Rzeszow, Poland; tomtrz@prz.edu.pl; 4 Department of Materials Science and Engineering of Non-Ferrous Metals, Faculty of Non-Ferrous Metals, AGH University of Krakow, Al. Adama Mickiewicza 30, 30-059 Cracow, Poland; mksiazek@agh.edu.pl; 5 Faculty of Materials Science and Engineering, Warsaw University of Technology, Woloska 141, 02-507 Warsaw, Poland; ryszard.sitek@pw.edu.pl; 6 Łukasiewicz Research Network—Krakow Institute of Technology, ul. Zakopiańska 73, 30-418 Cracow, Poland; adam.tchorz@kit.lukasiewicz.gov.pl; 7 Department of Geomechanics, Civil Engineering and Geotechnics, AGH University of Krakow, Al. Adama Mickiewicza 30, 30-059 Cracow, Poland; walach@agh.edu.pl

**Keywords:** casting, ceramic moulds, computed tomography, mould defects, porosity

## Abstract

This article presents the results of testing the suitability of X-ray computed tomography for the quality control of the casting moulds used for producing turbine blades. The research was focused on the analysis of cross-sectional images, spatial models and the porosity of moulds using a Phoenix L 450 microtomograph. The research material consisted of samples from three mixtures of ceramic materials and binders intended for producing casting moulds using the lost wax method. Various configurations of filling materials (Molochite and quartz flours) and binder (Remasol, Ludox PX 30 and hydrolysed ethyl silicate) mixtures were considered. X-ray computed tomography enabled the detection of a number of defects in the ceramic mass related to the distribution of mass components, porosity concentration and defects resulting from the specificity of the mould production. It was found that casting mould quality control on cross-sectional tomographic images is faster and as accurate as the analysis of three-dimensional models and allows for the detection of a whole range of ceramic defects, but the usefulness of the images is greatest only when the cross-sections are taken at an appropriate angle relative to the object being examined.

## 1. Introduction

X-ray computed tomography (CT), a non-destructive research method, is a versatile technique that can be used in many areas of industry [1]. Analysing the structure of materials is one of the applications of CT-based devices [2,3]. CT allows the analysis of a specific cross-section of a component or machine and its inspection for, for example, correct connections and missing parts, the detection of potential construction irregularities and the non-invasive checking of the internal defects of cast products. Industrial applications of X-ray CT are presented in paper [4].

A large number of production tests using CT equipment are qualitative analyses of fabricated cast products. Metallic castings are mainly characterised by defects in the form of porosity and voids [5,6], while cracks occur much more often in the case of hard materials such as ceramics. Defects in castings and foundry moulds are divided into four groups [7]: shape defects (on the outer surface of the casting), raw surface defects, material discontinuity (cracks) and internal defects. The main disadvantage of multilayer moulds are pores, which are empty spaces or defects in the structure of the material. To prevent the formation of porosity, the appropriate mould manufacturing technology should be prepared, as well as ensuring the appropriate parameters of the moulding sand [8,9].

Thanks to the X-ray CT method, it is possible to visualise defects in castings and moulds [10,11], perform geometric measurements of defects [12,13] or analyse the causes of the defects [14,15]. For mass production, it is also possible to create software that automatically defines defects in the context of compliance with applicable standards [16,17,18]. To improve the quality of product inspection for defects such as undesirable porosity in castings, investigations are carried out on the accuracy of measuring such defects using special reference objects as well as the selection of appropriate research equipment [19,20]. A large amount of research on porosity has been conducted in the context of improving the quality of ceramic products. Jerjen et al. [21], for example, analysed the influence of the shape and arrangement of pores inside samples taken from road asphalt on the absorption and evaporation of water. Haratym et al. [22] and Biernacki et al. [23] conducted research on ceramic casting moulds (CCMs) using a CT device to visualise the porosity and determine its percentage of the volume of the mould wall. Żaba et al. [24] used CT to assess the quality of spherical CCMs, taking into account cracks, porosity and changes in the thickness of the mould layers. Tchorz et al. [25] detected the inclusions and porosity in CCMs using X-ray CT technology. CT enabled the determination of the size of the pores and their exact location. In other articles, Tchorz et al. [26,27] proposed coupling CT with the physicochemical analysis of multilayer CCMs. Obtaining a virtual 3D model of the mould allowed the thickness of the mould layers and the porosity to be determined. Arunmuthu et al. [28] constructed 3D models of defects in bronze castings before high-cycle fatigue tests. The authors characterised casting defects according to size. They also concluded that the size of a defect varies as a function of the distance of the defect from the casting surface. Plessis and Rossouw [29] presented a case study on the rapid analysis of typical defects in titanium casting using the microCT method. They concluded that CT results for a component with a largest dimension of 225 mm can be obtained in less than 1 h. Bandara et al. [30] used dual-energy X-ray CT to evaluate the possibility of reducing porosity in castings using impregnated super sealant resins. They found that X-ray CT with a collimator creating fan beam is useful for the non-destructive characterisation of die castings.

In the literature, most works focus on the analysis of the porosity and defects in castings produced by various methods. Not much work has been devoted to the analysis of the defects in CCMs for producing turbine blades. Therefore, this article presents the results of testing the suitability of X-ray CT for the quality control of casting moulds manufactured using the lost wax method. The CCMs were made of various configurations of Molochite and quartz flours and binders. The research focused on the analysis of cross-sectional images, spatial models and the porosity of the casting moulds. A method for calculating the percentage porosity parameters of the samples based on the CT scans was proposed.

## 2. Materials and Methods

### 2.1. Material

The research material consisted of samples made of three mixtures of ceramic materials (Molochite and quartz flours) and binders intended for producing CCMs using the lost wax method. Ludox PX 30 (W. R. Grace and Company, Columbia, MD, USA), hydrolysed ethyl silicate and Remasol were used as binders. The tested casting moulds are used for casting turbine blades. Gradations of flours of 0.05–1 mm and 0.1–0.3 mm were used for the structural layer (SL) and the first two layers, respectively. Moulds with the material compositions included in the work are used to cast elements from iron alloys, aluminium, brass, and nickel-based superalloys. Each type of sample had seven layers of ceramic mass with different compositions. The first layer of the ceramic shell mould that is in contact with the liquid metal during the casting process deserves special attention. The material composition and properties of this layer are critical from the point of view of the cast product due to the direct contact with the metal. The components of individual layers of the three types of samples are presented in Table 1.

**Figure 1:**
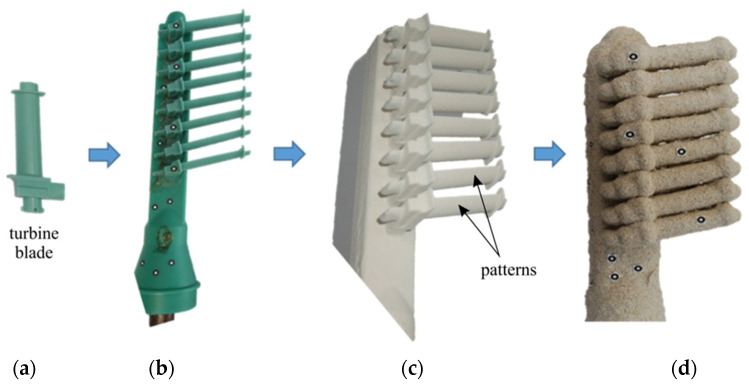
Scheme for producing research material in the form of CCMs: (**a**) wax model, (**b**) model set, (**c**) model set covered with the first ceramic layer, (**d**) casting mould.

**Figure 2:**
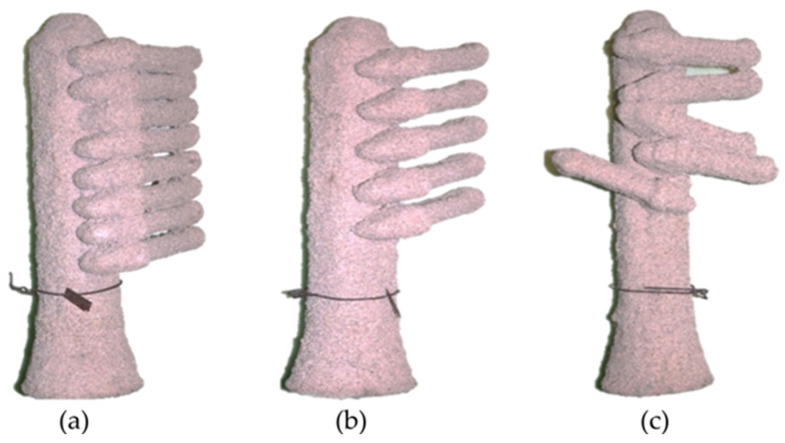
Casting mould shape samples: (**a**) F1, (**b**) F2, and (**c**) F3.

Samples are in the form of casting moulds with dimensions of 300 × 60 × 45 mm (not including the pouring basin) composed of a pouring system and cast part with dimensions of 120 × 35 × 25 mm, arranged in three different ways in order to observe the differences in the porosity distribution of the moulds between regular-shaped samples and highly complex samples. The samples were made by conventional production of moulds using the lost wax method (Figure 1), that is, wax patterns of the cast details were made and attached to the wax pouring system (depending on the sample, they contained from five to eight details). Layers of slurry were then applied and heat-treated to harden the mould and melt the wax from the sample. Figure 2 presents all types of analysed samples. Table 2 presents the notation assigned to the samples, which also contains information about the ingredients contained in the ceramic mass (Table 1).

### 2.2. X-Ray Tomography

X-ray tomographic examinations, depending on the type of samples, were carried out on a phoenix v|tome|x L 450 microtomograph (Jess W. Jackson & Assoc., Inc., 5018 Bristol Industrial Way Ste 206, Buford, GA 30518, USA) designed for examining large-sized objects. The v|tome|x L 450 tomograph is a versatile, high-resolution microtomograph enabling both 3D tomographic and 2D X-ray examinations. This class of microtomographs allows very large objects weighing up to 100 kg to be scanned. The system is equipped with a closed-type minifocus X-ray tube and a detector with contrast and resolution, enabling precise X-rays of industrial products. The research on the usefulness of CT for the quality control of casting moulds focused on the analysis of the cross-sectional images, spatial models and porosity. The operating parameters of the microCT v|tome|x L 450 tomograph used in the investigations are presented in Table 3. The principles of the testing and analysis of ceramic casting mould were the same for all tested mould samples of the same type. The aim of this process was to perform both a qualitative and quantitative assessment of ceramic casting moulds.

**Figure 3:**
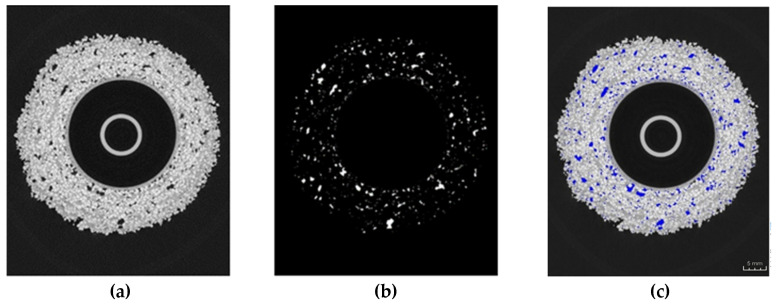
Methodology of porosity separation in a cross-sectional image: (**a**) image obtained from a tomograph, (**b**) porosity separation, (**c**) imposing porosity with a different colour to the original image [24].

Fiji Is Just ImageJ GPL v3+ 2.15.1 (FIJI) open-source image processing software was used to create a three-dimensional model of the mould with the porosity fraction in order to estimate the percentage of porosity in the sample. Figure 3 presents the process of separating the surface areas of porosity cross-sections inside the mould wall using the example of a spherical mould model. This procedure is intended to both apply colour to the areas of porosity in the cross-sectional images, which translates into easier visual analysis, and allow the 3D interpretation of the porosity inside the mass. For CCMs, it is necessary to manually define the centre of the mould in the programme as a large void, which is not, however, the porosity of the ceramic mass. For simple objects, for example, spheres, this is a simple procedure and can be automated. However, for objects with the complex geometry of a gating system, due to the irregularity of the centre of the mould relative to the central point of the image, the determination of porosity is difficult.

Three specialised programmes were used for the qualitative and quantitative analysis of the casting moulds: VGSTUDIO MAX 2024.2 (VGStudioMax), myVGL and FIJI, which enabled further processing, analysis and visualisation of data. To calculate porosity, tomographic sections were binarised and the colours of the pores were changed to be different from the colour of the ceramic mass. Next, the ratio of the number of pixels represented by the pores and the total number of pixels were determined. Spatial models of the examined objects based on the composition of each X-ray were created in the VGStudioMax (Volume Graphics) computer programme, which is dedicated to editing tomographic images.

The last stage of CT data processing was to visualise the porosity distribution inside the ceramic mould wall on spatial models. For this purpose, the myVGL programme was used, which is a free version of the VGStudioMAX software by Volume Graphics. The myVGL programme allows researchers to open projects created in the licenced version of VGStudioMAX and use selected tools. Thanks to this programme, it was possible to segment and classify different structures in CT data and realistically visualise three-dimensional models, which facilitated the interpretation and presentation of results. Colour filters were used to perform a qualitative analysis of porosity on spatial models of the moulds, which allowed for the isolation of the density of individual components of the ceramic mould.

## 3. Results

In this section a method of inspecting cross-sectional images of samples for potentially occur-ring material defects as well as spatial models reconstructed on this basis is presented. In addition, quantitative CT results and the shortcomings of qualitative CT results are pre-sented.

### 3.1. Cross-Sectional Images

**Figure 4:**
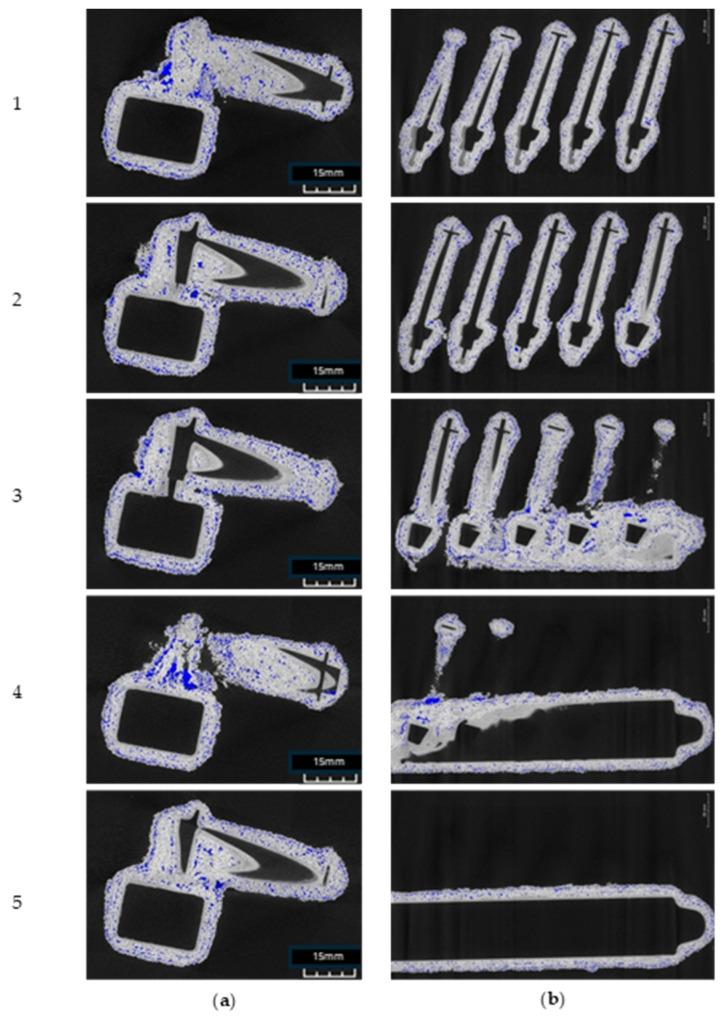
Cross-sectional X-ray tomographic images of the FM2 casting mould in planes (**a**) parallel and (**b**) perpendicular to the mould detail.

**Figure 5:**
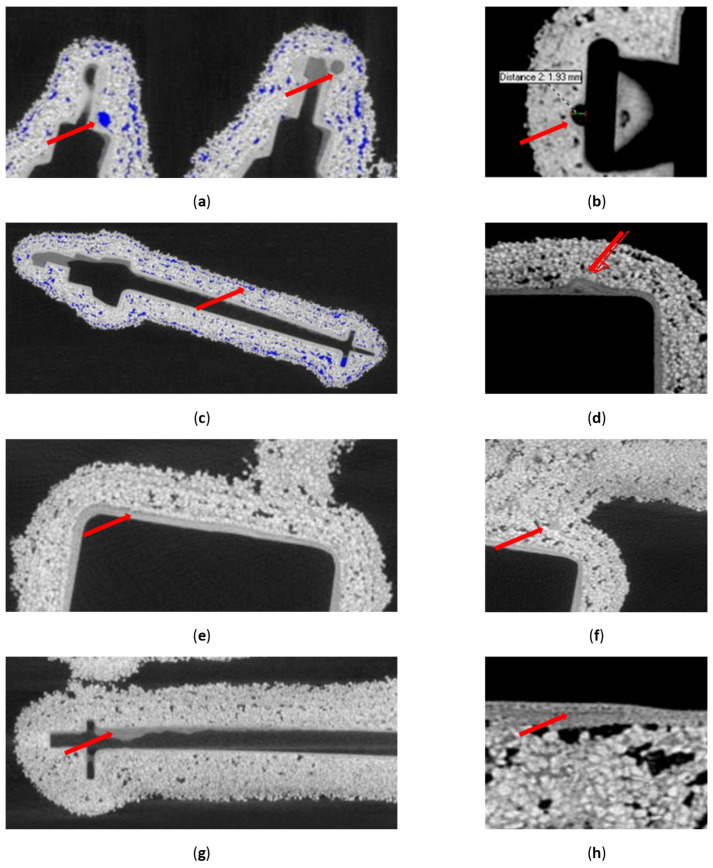
Defined defects of the tested moulds: (**a**) voids between the first layer and the structural layers; (**b**) distortion of the first layer; (**c**) layered distribution of porosity; (**d**) uneven distribution of the material of the first layer; (**e**) delamination of the mould material; (**f**) binder concentration; (**g**) wax residues and (**h**) fine porosity inside the first layer.

Figure 4 presents cross-sectional tomographic images of a casting mould. In the first image (Figure 4a), 971 X-ray overexposures were taken for sample FM2, and in the second image (Figure 4b), 449 X-ray overexposures were taken. Five cross-sectional images of sample FM2 with five details and a gating system were selected in such a way as to first capture each detail individually in a plane parallel to the details, and secondly to visually inspect the cross-sectional images in a second plane covering all the details and the entire gating system at the same time. The number of X-ray overexposures in each of the two projections of the rest of the forms varied depending on their exact overall dimensions. Figure 5 presents a number ofdefects identified by analysing cross-sectional images of the casting moulds. Voids between the structural layers and the first layer (Figure 5a), the distortion of the first layer (Figure 5b) and the uneven distribution of the first layer material (Figure 5d) were noticed. In the structural layer, a layered distribution of porosity (Figure 5c), the delamination of the mould material (Figure 5e), binder concentration (Figure 5f), binder residue (Figure 5g) and fine porosity inside the model layer (Figure 5h) can be noticed.

### 3.2. Three-Dimensional Models

**Figure 6:**
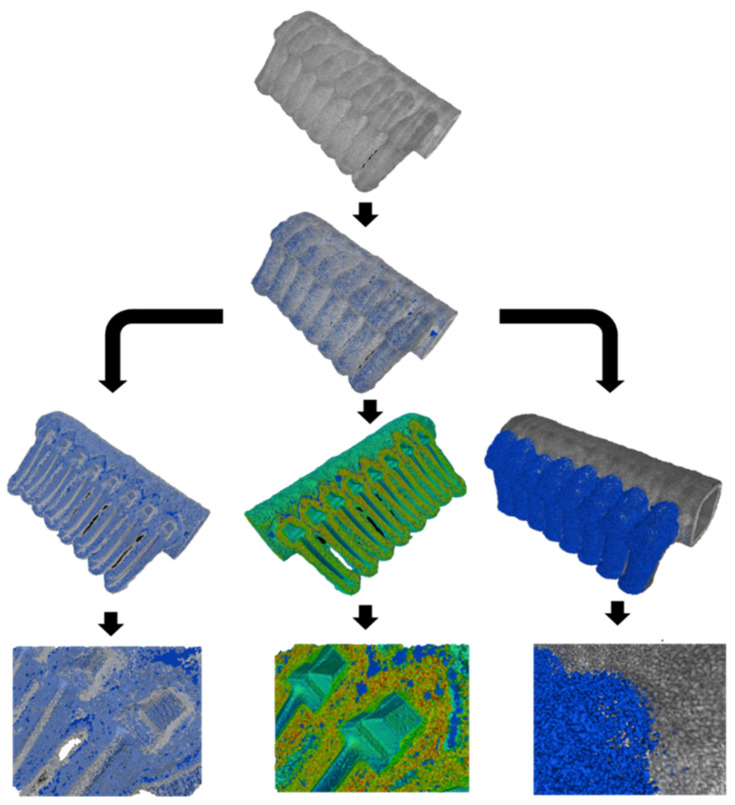
The myVGL programme filters highlighting the distribution of porosity and components of the casting mould wall.

**Figure 7:**
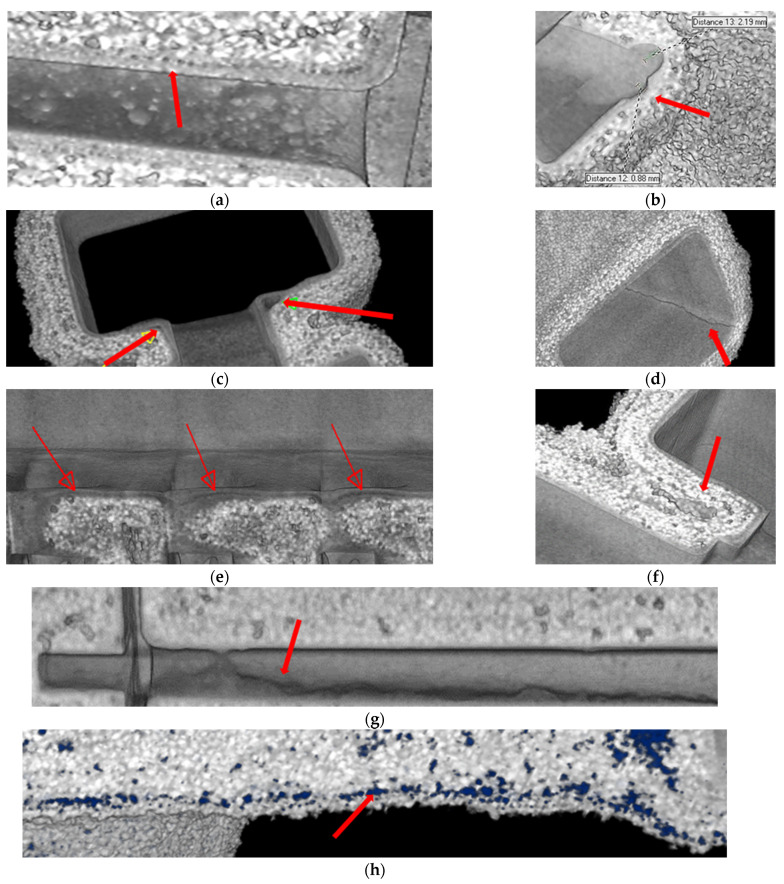
Defined defects of the tested moulds: (**a**) open porosity; (**b**) mismatch between the first layer and the wax model; (**c**) voids between the first layer and the structural layer; (**d**) local crack in the first layer; (**e**) local delamination; (**f**) large void; (**g**) wax residue; (**h**) layered concentration of porosity.

Figure 6 shows a diagram of the use of three functions of the myVGL programme that applied filters to the reconstructed three-dimensional model of the FM1 ceramic casting mould, enabling the detection of defects based on the density distribution of individual ceramic components or the distribution of porosity inside the wall. The inspection of these consisted in applying reconstructed porosity (blue colour in Figure 6) to the mould model. Then, for the purposes of analysis, a filter was applied to the mould model, highlighting (from left to right), the semi-transparency of the mould material, the density of the porosity components, and a local combination of the porosity and the mould material. Various defects in the sample material identified using the 3D models are presented in Figure 7. In the first layer, a mismatch of this layer with the wax model (Figure 7b), voids between the first layer and the structural layer (Figure 7c) and a local crack in the first layer (Figure 7d) were identified. The structural layer of the mould was characterised by open porosity (Figure 7a), local delamination (Figure 7e), large voids (Figure 7f), wax residue (Figure 7g) and layered porosity concentration (Figure 7h).

### 3.3. Percentage Porosity

**Figure 8:**
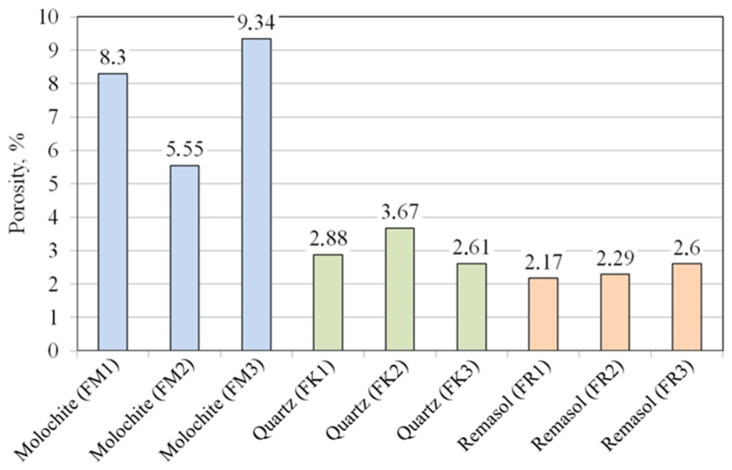
Percentage porosity of the casting moulds.

Figure 8 shows the percentage porosity determined by CT for three series of samples. The samples with Molochite as the filling material were characterised by the highest porosity (5.55–9.34%). However, the porosity value for these samples was strongly related to their shape (Figure 2). Samples filled with quartz flour and Remasol were characterised by much lower porosity and this was similar in all analysed mould shapes. Remasol provided the lowest porosity, 2.17% and 2.6% for samples F1 (Figure 2a) and F3 (Figure 2c), respectively.

### 3.4. Summary of the CT Results

Table 4 shows all the defects in each of the test sample that were identified by analysing cross-sectional images and three-dimensional spatial models. The following defects correspond to the designations in the table: 

Crack in the structural layer;

Crack in the first layer;

Mismatch between the first layer and the wax model;

Local delamination of the first layer and structural layer;

Foreign body of high density;

Porosity concentration between the first structural layers;

Porosity concentration between the middle structural layers;

Porosity concentration between the last structural layers;

Porosity concentration between the first layer and the structural layer;

Porosity concentration—large fraction between structural layers;

Porosity concentration between any layers;

Heterogeneity of porosity distribution throughout the sample volume;

Local binder concentration;

Open porosity;

Large amounts of porosity in the first layer;

Wax residue inside the sample.

**Figure 9:**
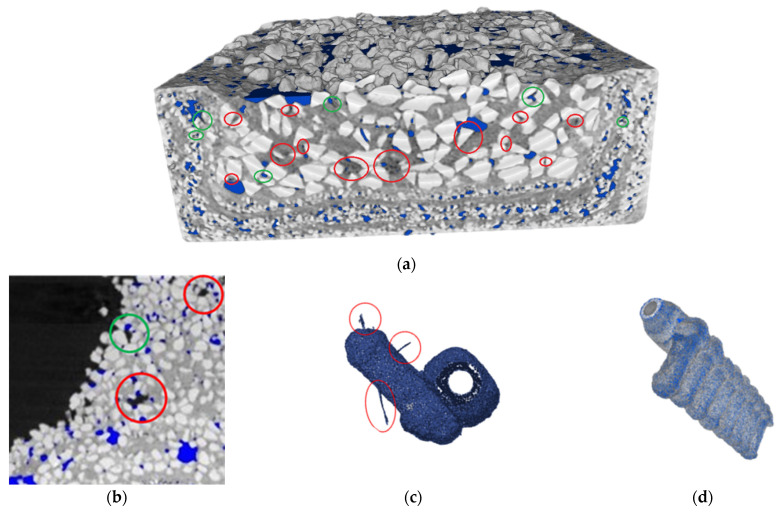
Disadvantages of digital CT examination results: (**a**) mismatch of localised porosity with the dimensions of the void (green area) or its omission (red area) in the three-dimensional model; (**b**) mismatch of localised porosity with the dimensions of the void in the cross-sectional images or its omission; (**c**) artefacts and (**d**) unreal porosity on the surface of the spatial model of the mould.

Figure 9 presents the defects in the processing images and the defects in the reconstruction of the 3D models of the research material identified during the analysis of the results:

- Mismatch of the localised void (blue area) to its geometry;
- Omission of the void, which can be detected at the given spatial resolution of the tomograph;
- Spatial image artefacts—solid bodies that do not exist in reality (fragments of the tested sample);
- Generated porosity that is not actually present in the sample (the defect is present on the external and internal surfaces of the samples).

**Figure 10:**
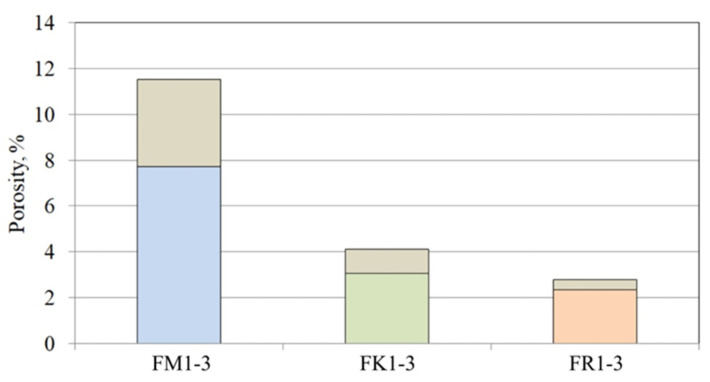
Statistical features of percentage porosity obtained by CT.

Figure 10 statistically presents the percentage porosity parameter depending on the ceramic mixture used for each series of tested samples, as well as the estimated stability of the parameter for a given mixture.

## 4. Discussion

### 4.1. Defects Obtained by CT

All components of the ceramic mass, that is, binder and filler material, as well as porosity inside the mould wall and other foreign bodies, were identified in three series of samples. Both cross-sectional X-rays and reconstructed three-dimensional models obtained using a microCT tomograph provided multi-sectional qualitative information about moulding materials, in particular information about the following:

- Defects in the first layer, that is, distortions resulting from the presence of air bubbles between the wax and the first layer, open porosity, local detachment of the first layer from the structural layers, local location of the material of the first layer in the structural layer, cracks and large-diameter porosity;
- Defects in structural layers: cracks, concentration of porosity, large pores and voids, foreign bodies and delamination of structural layers;
- Binder defects: binder concentration (with a simultaneous lack of filler grains), layered binder concentration and binder porosity.

It should be emphasised that defining defects in the analysed moulds was much more reliable, and often only possible, when comparing tomographic results of individual sections and the spatial model. When a defect was found on one of the cross-sectional images, it was also possible to trace the location of the defect in adjacent cross-sections in a very short time. Additionally, it was possible to better visualise the area of interest using a spatial model, based on the number of cross-sectional images and the defined cross-sectional axis.

A filter distinguishing the binder material, filler and porosity of the test material, also called the semi-transparency filter of the moulding sand, made it easier to locate a concentration of one of the components or defects in the form of voids. Anomalies defined and located in this way were easier to find and mark in the next stage on the cross-sectional tomographic images, which, due to the contrast and grey scale, were often better suited for reporting defects found in the samples.

Thanks to three types of arrangement of blade-shaped details, it was possible to check their influence on the porosity concentration and the formation of voids between the mould components. Analysis of the shape of the samples in the tomographic images proved that there is a relationship between the local geometry of the blade and the porosity concentration or the presence of large voids inside the wall of the ceramic mould. Regardless of the type of ceramic mass mixtures used, the local presence of the wall morphology mentioned was noticeable on the part of the mould perpendicular to the airfoil of the blade. Some of the samples had defects that could cause the improper solidification of the metal during pouring as a result of uneven heat dissipation, which would potentially lead to the cast turbine blades having an undesirable microstructure.

### 4.2. Percentage Porosity of Samples Determined by CT

The FM1–FM3 series of moulds had a percentage porosity that was more than twice as high compared to the other samples. This is due to the concentration of porosity in the last layer, which, as opposed to the remaining series of material forms, covers a large space of each detail. The remaining series had a porosity similar to each other for each type of detail arrangement (Figure 2). At the same time, there was no observed effect of the distance of the details from each other on the porosity of the moulds, despite the presence of large voids between closely located patterns (Figure 1), qualified during the thresholding process to expose the porosity. It is also worth noting that the porosity range (Figure 10) in the FM1–FM3 samples compared to the FK1–FK3 and FR1–FR3 series was much larger, which may suggest limited control over porosity for this series of materials and shape of moulds.

The percentage porosity of the samples, despite the significant lack of precision resulting from the data collection method, clearly differentiates the samples within one series as more and less porous, due to both different component materials and sample geometry. A significant part of the pore surface of a specific fraction, which differs between samples within one material group, is within the inspection capabilities of the microCT tomograph. In particular, large pores and voids have the greatest impact on the diversity of the morphology of the ceramic wall and are directly related to heat dissipation.

### 4.3. Errors in Porosity Localisation and Reconstruction of Spatial CT Models

The analysis of the CT results revealed two types of errors occurring during the processing of the results. The reconstructed spatial models have artefacts, the formation of which may be related to the attachment of supporting elements, ensuring an appropriate angle in relation to the instruments of the CT device. These artefacts did not affect the qualitative analysis, they were not inside the mould wall, and removal was possible thanks to the voxel separation functions of a specific density in the myVGL programme.

The mismatch of the porosity defined during the thresholding of cross-sectional tomographic images (blue area in Figure 9a) results from how the image transformation operates. Due to the different shades of grey in the image, when averaging these colours (binder and filler material of the sample wall), a local change in the colour of the pixel may occur. Then, after separating the background colour from inside the wall (porosity—air) and re-applying it to the output CT image, deviations from the actual shape of the void are noticeable. However, these are sporadic cases and the scale of mismatch compared to the amount of porosity is very small.

Similarly, the porosity identified on the surface of 3D models, which does not actually occur, is the result of the programme’s inability to distinguish the cross-sectional image from the voids between the grains, which constitute the actual void inside the examined object. However, the voids existing in the cross-section which pass through fragments of the mould located close to the mould surface do not represent actual voids inside the mould wall.

### 4.4. Limitations of CT Devices

The dimensions of the examined object are a factor determining the quality of the CT scans performed. Differences in the size of the spatial resolution parameter (voxel) occur depending on the arrangement of the details. At the same time, it should be noted that industrial casting moulds, depending on the size of the cast element itself, can reach much larger dimensions than those presented in this article. Scans of such research material will have a much more visible loss of quality compared to small elements, limiting the possibility of detecting defects and porosity in a small fraction.

The second important factor limiting the use of CT to assess the quality of ceramic moulds is the test time. The total time of the test performed on the analysed ceramic moulds consists of a number of time-consuming operations: calibration of the CT device (up to several hours); X-ray exposure of the sample (approx. 3 h); processing of the cross-sectional images (approx. 2 h); post-processing of the cross-sectional CT images (from 3 to 10 h depending on the sample geometry); reconstruction of the spatial model (up to several hours); and analysis of the results for any defects (up to several hours). Depending on the type of sample, individual times vary, primarily the reconstruction times of the spatial model of samples of various shapes, even within one series, and the analysis time for each sample.

## 5. Summary and Conclusions

Research into the possibility of using a microtomograph to assess the quality of ceramic casting moulds produced using the lost wax method resulted in the creation of a comprehensive set of knowledge on the examined issue, which may be used to facilitate decisions regarding the implementation of this type of device in dedicated production plants. It was proven that computed tomography enables the detection of a number of ceramic mass defects related to the distribution of mass components, porosity concentration and defects resulting from the specificity of mould production. The justification for the usefulness of the spatial model editing function of the analysed software for detecting and reporting sample defects was presented.

The following conclusions are the result of the research conducted:

- CT enables the detection of a number of defects in the ceramic mass related to the distribution of mass components, porosity concentration and defects resulting from the specificity of the mould production;
- The FM1–FM3 series of moulds containing Molochite flour as a filling material was characterised by a percentage porosity that was more than twice as high compared to other samples; this is due to the concentration of porosity in the last layer, which, as opposed to the remaining series of moulds, covers a large space in each mould;
- The presented method of calculating the sample’s percentage porosity, despite imperfections, reliably differentiates samples in terms of the presence of pores, and the accuracy of the microCT device is sufficient to be a source of information about the permissible or impermissible percentage of pores in the ceramic mass;
- The quality control of CCMs on cross-sectional CT images is faster and as accurate as the analysis of spatial models and defines a whole range of ceramic defects, but the usefulness of the images is greatest only when the cross-section angle of the image is appropriate in relation to the object being examined;
- The analysis of casting moulds in the variants presented in the article is definitely too time-consuming from an industrial point of view, but it can be a good control tool for new, experimental batches of moulds with a new ceramic mass composition and different geometry.

## Figures and Tables

**Table 1:** Components of the ceramic mixtures used to make the samples.

Type	Name	Number of Layers	Binder	Filling Material
1	Molochite	1 2	Ludox PX 30 and Molochite flour hydrolysed ethyl silicate and Molochite flour	Molochite sand (0.1–0.3 mm)
		3 4–7	Ludox PX 30 and Molochite flour alternating layers of Ludox PX 30 and hydrolysed ethyl silicate	Molochite sand (0.5–1 mm)
2	Quartz	1 2	hydrolysed ethyl silicate hydrolysed ethyl silicate and quartz flour	Quartz sand (0.1–0.3 mm)
		3 4–7	Ludox PX 30 and quartz flour alternating layers of Ludox PX 30 and hydrolysed ethyl silicate	Quartz sand (0.5–1 mm)
3	Remasol	1–2	Remasol Plus and quartz flour	Quartz sand (0.1–0.3 mm)
		3–7	Remasol Premium and quartz flour	Quartz sand (0.5–1 mm)

**Table 2:** Notation of samples.

Name of the Ceramic Mixture	Notation
Molochite (M)	FM1, FM2, FM3
Quartz (K)	FK1, FK2, FK3
Remasol (R)	FR1, FR2, FR3

**Table 3:** Test parameters of the microCT v|tome|x L 450 tomograph.

Parameter	Value
Primary X-ray source	200 kV
Current intensity I	300 µA
Exposure time	500 ms
Number of exposures	2200
Voxel size	123.6 µm
Type of lamp	microfocus 300 kV
Filter	0.5 mm thick copper (Cu) filter

**Table 4:** Defects in the ceramic samples with various composition.

Sample	Defect															
	1	2	3	4	5	6	7	8	9	10	11	12	13	14	15	16
FM1	✔		✔					✔						✔		
FM2	✔		✔					✔								
FM3			✔					✔						✔		
FK1				✔					✔	✔		✔			✔	
FK2			✔								✔		✔			
FK3	✔		✔	✔								✔				
FR1			✔			✔						✔		✔		✔
FR2			✔			✔				✔						
FR3			✔									✔	✔			

## Data Availability

Data are available from the first author and can be shared with anyone upon reasonable request.
